# BaBao Dan Suppresses Tumor Growth of Pancreatic Cancer Through Modulating Transcriptional Reprogramming of Cancer-Related Genes

**DOI:** 10.3389/fonc.2020.584330

**Published:** 2020-11-19

**Authors:** Libin Song, Zhixiao Fang, Chuanfang Pan, Xiangyuan Chen, Xiang Qian, Yunyun Cai, Xiumei Zhang, Luming Liu

**Affiliations:** ^1^ Department of Integrative Oncology, Fudan University, Shanghai Cancer Center, Shanghai, China; ^2^ Department of Oncology, Shanghai Medical College, Fudan University, Shanghai, China; ^3^ Institute of Translational Medicine, Shanghai General Hospital, Shanghai Jiao Tong University School of Medicine, Shanghai, China; ^4^ Department of Oncology, Longhua Hospital affiliated to Shanghai University of Traditional Chinese Medicine, Shanghai, China; ^5^ Department of Geriatrics, Huadong Hospital, Shanghai Medical College, Fudan University, Shanghai, China; ^6^ Institute of Cancer and Basic Medicine (ICBM), Chinese Academy of Sciences, Zheijang, China; ^7^ Department of Traditional Chinese Medicine, Zhejiang Cancer Hospitall, Zheijang, China; ^8^ Department of Integrated Traditional Western Medicine, Minhang Branch of Fudan University of Shanghai Cancer Center, Shanghai, China

**Keywords:** Babao Dan, pancreatic cancer, enrichment analysis, metabolism, transcriptome

## Abstract

**Background and Aims:**

Pancreatic ductal adenocarcinoma (PDAC) is one of refractory malignancies without efficient therapeutics. Babao Dan (BBD) was partially effective to suppress tumor growth of PDAC in clinical practice. However, the molecular mechanisms were unclear.

**Methods:**

We established PDAC mice models and treated them with BBD through intragastric administration. Treatment and control groups were then subjected to high-throughput RNA sequencing. We presented the transcriptional changes upon BBD treatment by using computational analysis comparing BBD treatment and control groups. Functional enrichment analysis was employed to investigate the biological processes or pathways that BBD modulates.

**Results:**

BBD treatment showed strong suppression on tumor growth of PDAC, even stronger than Gemcitabine. Through differential analysis comparing BBD treatment and control groups, we identified 638 up-regulated and 259 down-regulated genes in the BBD treatment group. BBD was found to activate tumor suppressor genes, such as MTUS1, PDGFB, SOD3, and UCHL1. Furthermore, we revealed that BBD treatment inhibited cancer-related pathways and elevated activities of metabolism-related processes. The BBD-modulated metabolic genes were further showed to be associated with patient survival in an independent cohort with pancreatic cancer.

**Conclusion:**

BBD repressed the tumor growth of PDAC. BBD treatment modulated expression of cancer-related genes in PDAC. BBD suppressed cancer-related pathways and activated metabolic processes in PDAC. Our study suggests BBD treatment as potential effective therapeutics for patients with pancreatic cancer.

## Introduction

Pancreatic ductal adenocarcinoma (PDAC) is the most common malignancy of thepancreas, which is among the leading causes of cancer-related death (1). With a 5-year survival rate of ~5% and a median survival duration less than 11 months, PDAC has the highest incidence-to-mortality ratio among solid tumors (2). Although substantial improvements of understanding the development and progression of PDAC have been made, few scientific advances have been translated into effective clinical therapies. Therefore, it is of tremendous need to develop novel therapeutic strategies and discover efficient drugs for PDAC (3).

Babao Dan (BBD) is a mixed powder of traditional Chinese medicine with potential antifibrotic, immunomodulatory, and antineoplastic activities, which mainly contains natural calculus bovis, snake gall, antelope horn, pearl, musk, and radix notoginseng. BBD is well known to improve liver function and is widely used as a complementary and alternative medicine to treat chronic liver disease in China and East Asian countries (4). Furthermore, BBD has been demonstrated to have significant anti-tumor effects. In particular, BBD was reported to induce cell apoptosis of gastric cancer through regulating MAPK and NF-*κ*B signaling pathways (5). Liu et al. revealed that BBD was able to inhibit the gastric cancer cell migration and invasion *via* suppressing epithelial–mesenchymal transition (EMT) (6). BBD was found to be partially effective for patients with pancreatic cancer in clinical practice. Further investigation on underlying mechanism is needed to optimize therapeutic utilization of BBD for PDAC patients. Computational analysis on cancer transcriptome has been revealed to discover key genes or pathways that were dysregulated during cancer progression (7, 8).

To further understand the anti-tumor mechanisms of BBD in PDAC, we established a PADC mouse model. High-throughput RNA sequencing technologies were employed to recapitulate transcriptional changes upon BBD treatment. We identified 897 differential genes in the BBD treatment group compared with the control group. The BBD down-regulated genes were revealed to be enriched in cancer-related pathways, while up-regulated genes were enriched in metabolic processes. Our study characterized transcriptional reprogramming upon BBD treatment in PDAC, presenting the possible molecular mechanism that BBD treatment suppressed tumor growth of PDAC.

## Materials and Methods

### Cell Culture

Human PDAC cell line Panc1 was purchased from the Type Culture Collection of the Chinese Academy of Science (Shanghai, China). Dulbecco’s modified Eagle’s medium (DMEM, Gibco; Thermo Fisher Scientific, Inc.) was employed to maintain Panc1 cells with 100 units/ml penicillin, 100 mg/ml streptomycin, and 10% fetal bovine serum (Gibco; Thermo Fisher Scientific, Inc.). Cells were cultured at 37°C in a humidified atmosphere containing 5% CO_2_.

### *In Vivo* Assays

Female nude mice, aged 5–6 weeks old, were purchased from the Experimental Animal Center of the Shanghai Cancer Institute (Shanghai, China). All mice were divided into five groups with 10 mice in each group, including Gemcitabine group, three BBD treatment groups and control group. Each mouse was subcutaneously injected through axilla with Panc1 cells (1 × 10^7^) that were suspended in 0.2 ml serum-free DMEM. After 3 weeks of tumor growth, mice were treated accordingly in the different groups. Mice in the control group were treated with saline (0.2 ml) through intraperitoneal injection. The Gemcitabine group was intraperitoneally injected with Gemcitabine (0.2 ml). BBD treatment groups were treated through intragastric administration with BBD (0.2 ml) of 0.125 g/kg, 0.25 g/kg, and 0.5 g/kg dose, respectively. All these treatments were conducted in days 1, 5, and 9. The tumor growth was monitored, and mice were sacrificed after 4 weeks from the first treatment. Tumor sizes and weights were recorded twice every week.

### RNA Sequencing

The TRIzol reagent (Invitrogen) was employed to collect total RNA samples (3 μg), which was then treated with the RiboMinus Eukaryote Kit (Qiagen, Valencia, CA) to remove rRNA. To prepare strand-specific RNA sequencing libraries, the NEBNext Ultra Directional RNA Library Prep Kit for Illumina (NEB, Beverly, MA) was used according to the manufacturer’s instructions. In brief, random hexamer primers were used to synthesize first- and second-strand complementary DNA (cDNA) from the fragmented ribosome-depleted RNA samples (50 ng). In consideration of removal of the second strand, the second-strand cDNA was synthesized by using dUTP mix. The ends were repaired by treating cDNA fragments with the End-It DNA End Repair Kit, then cDNA fragments were modified with Klenow to add an adenosine at the 3′-end, and finally were ligated to the adaptors. The second-strand cDNA was removed from the ligated cDNA products by purifying and treating with uracil DNA glycosylase. Purified first-strand cDNA was subjected to 12–15 cycles of PCR amplification, and the libraries were quality-controlled with a Bioanalyzer 2100 (Agilent, Santa Clara, CA) and sequenced by HiSeq 2000 (Illumina, San Diego, CA, USA). The raw sequencing reads were deposited in the Gene Expression Omnibus (GEO) database under accession GSE138963.

### Processing RNA-Seq Data

The Trimmomatic (Version 0.36) software (9) was adopted to clip adapter sequences and low-quality bases from the raw sequencing reads with default parameters. Then, all filtered reads were aligned to the human reference genome (GRch38) by using the splice-aware aligner HISAT2 with default settings (10). Afterwards, the alignments were subjected to StingTie program (11) to evaluate the gene expression levels in TPM units (TPM = Transcripts Per Million mapped reads). The GENCODE gene annotation (release v28) was employed to quantify the gene expression.

### Identification of lncRNA-Associated Protein-Coding Genes

For each candidate lncRNA, Spearman correlations between expression of lncRNAs and all individual protein-coding genes were calculated. Genes with Benjamini–Hochberg adjusted P values <0.05 were ranked by coefficients for each lncRNA. The top 1% rank protein-coding genes of individual lncRNAs were defined as the lncRNA-associated protein-coding genes and used for following enrichment analysis.

### Differential Expression Analysis

The DESeq2 software (12) was employed to refer to differentially expressed genes by using the raw counts of all genes with detected expression in at least one group. Genes with fold change no less than 1.5 and p value less than 0.01 were regarded as significantly differential genes in BBD treatment group compared with the control group. In addition, the differentially expressed genes of the TCGA PAAD cohort were obtained from the GEPIA2 database (http://gepia2.cancer-pku.cn), which identified differential genes by using ANOVA method (13).

### Western Blotting Assays

MUTUS1 and UCHL-1 proteins were separated and transferred to nitrocellulose membranes (Bio-Rad, Hercules, CA, USA), which were blocked with 5% non-fat milk and incubated with corresponding primary antibodies (AATF, AKT-s473, AKT, and PD-L1) followed by horseradish peroxidase-conjugated secondary antibodies. SDS-PAGE was used to conduct protein separation and transfer. Then, the chemiluminescence and enhanced chemiluminescence (ECL) reagents (Pierce Biotechnology, Rockford, IL, USA) were utilized to visualize the immunoreactivity, and the densitometry was measured using the Image-Pro Plus 6.0 (Media Cybernetics, Rockville, MD, USA).

### Functional Enrichment Analysis

The list of unique protein coding genes or long non-coding RNA associated genes was used to determine the enrichment in Gene Ontology (GO) biological processes or KEGG pathways. The gene sets of GO biological processes and KEGG pathways were retrieved from the Molecular Signature Database (MSigDB) (14). A hypergeometric test was performed to calculate the enrichment significance. The probability P was computed to assess the enrichment significance as follows (8):

$$\begin{array}{l} \text{P}=1-\text{F}(x|N,K,M)\\ \;=1-\sum_{t=0}^{x}\frac{{(}_{t}^{K}){(}_{M-t}^{N-K})}{{(}_{M}^{N})} \end{array}$$

where *N* is the total number of GENCODE release v28 protein-coding genes, *K* indicates the number of genes in the sets of GO biological processes and KEGG pathways under investigation, *M* represents the number of protein-coding genes for analysis, and *x* is the number of genes overlapped between interested protein-coding genes and investigated GO terms or KEGG pathways.

### Gene Set Enrichment Analysis

The differentially expressed genes (DEGs) or differential lncRNA-associated protein-coding genes were ranked by fold-change values. The pre-ranked lists were then subjected to the Gene Set Enrichment Analysis (GSEA) algorithm (15) implemented in R package clusterProfiler (16) to investigate the significantly enriched GO biological processes or KEGG pathways.

### Survival Analysis in TCGA PAAD Cohort

The clinical data of the TCGA PAAD cohort were retrieved from the Genomic Data Commons Data Portal (GDC, https://portal.gdc.cancer.gov/) (17). For each DEG, the “survival” R package was employed to compare the survival difference between different expression groups. Particularly, patients were divided into two groups according to expression of corresponding genes with median values as cutoff. Then “Surv” function was used to construct a surv object based on the vital status and survival time of patients in different groups. The fitted survival curves were created by using function “survfit”, wherein the Kaplan–Meier algorithm was implemented based on the above surv object and group labels. Finally, log-rank test was employed to compare the difference between these two survival curves. Genes with P value <0.05 were regarded to be significantly associated with overall survival of patients with pancreatic cancer. Furthermore, gene expression changes and survival association were also calculated in the PACA-AU cohort from ICGC project (https://dcc.icgc.org/releases/release_25/Projects/PACA-AU). We showed HSD17B14 as an example in **Supplementary Figure 8**.

### Statistical Analysis

All statistical analysis and data visualization in the present study were performed by using R software (http://www.r-project.org). Unless specifically stated, all tests were two-tailed and P < 0.05 was considered to be statistically significant.

## Results

### Babao Dan Significantly Suppresses Tumor Growth of PDAC

**Figure 1:**
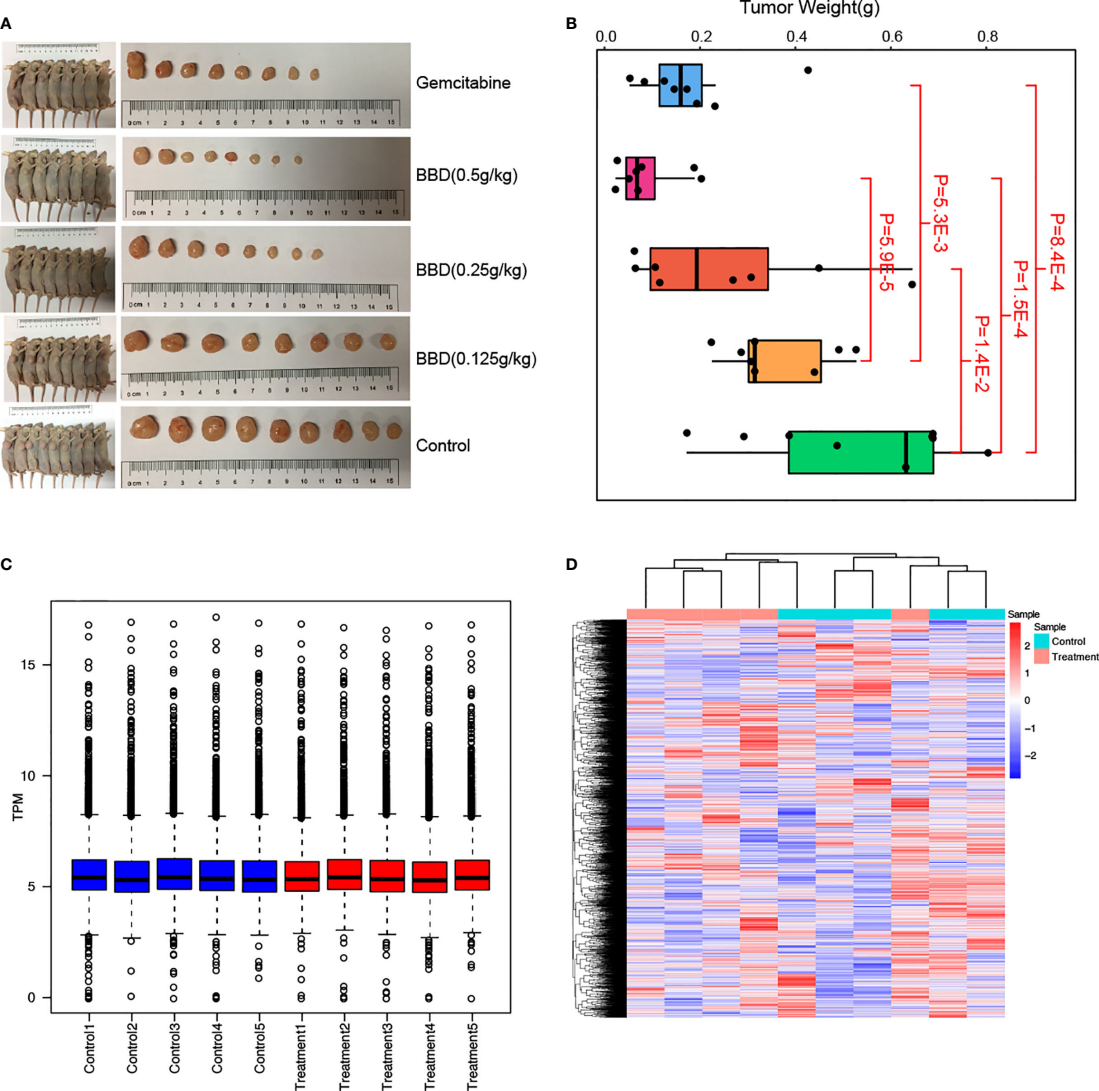
BBD significantly suppress tumor growth of PDAC. **(A)** Comparison of tumor sizes in Gemcitabine, 0.5 g/kg dose BBD, 0.25 g/kg dose BBD, 0.125 g/kg dose BBD, and control mice groups. **(B)** Comparisons of tumor weight between different mice groups. Correlations of gene expression between different samples in control **(C)** and 23.2 g/kg dose BBD mice groups **(D)**.

To explore the underlying molecular mechanisms of BBD on pancreatic cancer, we utilized different doses of BBD to treat pancreatic cancer mice models (see *Materials and Methods*). The tumor volumes and weights were significantly decreased in BBD treatment mice groups than those in the control group (**Figures 1A, B** **)**. Larger doses of BBD had stronger suppression effect on tumor growth of PDAC (**Figure 1B**). Furthermore, treatment of 0.5 g/kg dose BBD showed stronger tumor suppression power than Gemcitabine, which has been an effective chemotherapy for PDAC patients (18). To further investigate underlying molecular pathways or key genes that BBD may interfere, we performed high-throughput RNA sequencing of samples from 0.5 g/kg dose BBD treatment and control groups. Normalized read counts of all detected genes were used to perform correlation analysis between different samples in the same groups. High correlation coefficients were observed between different samples within the same groups (0.964 in control group and 0.962 in BBD treatment group, in average, **Supplementary Figure 1**). This observation showed the high-reliability and high-quality of collected samples and RNA sequencing in our study. The distribution of gene expression showed overall consistence among different samples, suggesting reliability of our data (**Figure 1C**). Furthermore, clustering analysis showed the difference of expression between different sample groups (**Figure 1D**). The exception of one treatment sample may be due to the heterogeneity of response to BBD treatment. Our study showed that BBD is able to suppress tumor growth of PDAC, suggesting BBD treatment as alternative therapeutics for patients with pancreatic cancer.

### PDAC-Associated Genes Were Dysregulated by Babao Dan Treatment

**Figure 2:**
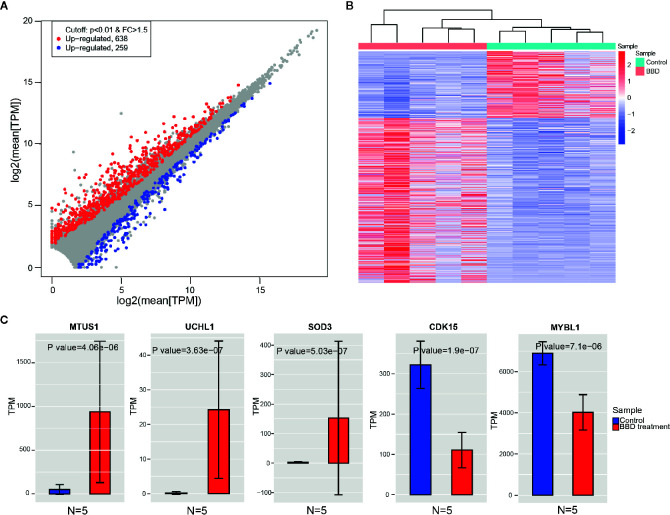
Changes of gene expression between BBD treatment and control groups. **(A)** Comparisons of gene expression in BBD treatment and control groups. X-axis indicates log2 transformed normalized read counts in control groups, and Y-axis is BBD treatment group **(B)** Heatmap shows significantly differentially expressed genes between BBD treatment and control groups. **(C)** Comparisons of gene expression of MTUS1, UCHL1, SOD3, CDK15, and MYBL1 between BBD treatment and control groups.

In order to identify the genes that were affected by BBD treatment, we compared the gene expression between BBD treatment and control groups. Consequently, 638 genes were found to be up-regulated, and 259 genes were down-regulated in BBD treatment group (**Figures 2A, B** **)**. Most of the DEGs were protein-coding genes (659 mRNAs, 74% of all DEGs, **Supplementary Figures 1A, B**). In addition, over half of the differential non-coding genes were long non-coding RNAs (lncRNA) (139 lncRNAs, 59% of all differential non-coding genes, **Supplementary Figures 1C, D**). Among these DEGs, several tumor suppressor genes were found to be up-regulated in BBD treatment group, such as MTUS1 (19), SOD3 (20), and UCHL1 (21) (**Figure 2C**). The protein changes of MTUS1 and UCHL1 were validated by Western blotting (**Supplementary Figure 4**). BBD may activate these tumor suppressors to inhibit tumor growth. For example, re-expression of SOD3 was reported to diminish the invasiveness and growth of pancreatic ductal adenocarcinoma (20). These results indicate that BBD treatment might repress tumor growth of PDAC by up-regulating tumor suppressor genes. Furthermore, some oncogenes were inhibited by BBD treatment, such as CDK15 and MYBL1. BBD treatment was also found to modulate the expression of lncRNAs, such as LINC02223 (**Supplementary Figure 3**). Additionally, genes with substantial expression changes upon BBD treatment may be possible targets of BBD (**Supplementary Figure 3**). In summary, BBD treatment suppresses tumor growth of PDAC through modulating tumor-related genes.

### BBD Treatment Inhibits Cancer Pathways and Activates Metabolic Processes

**Figure 3:**
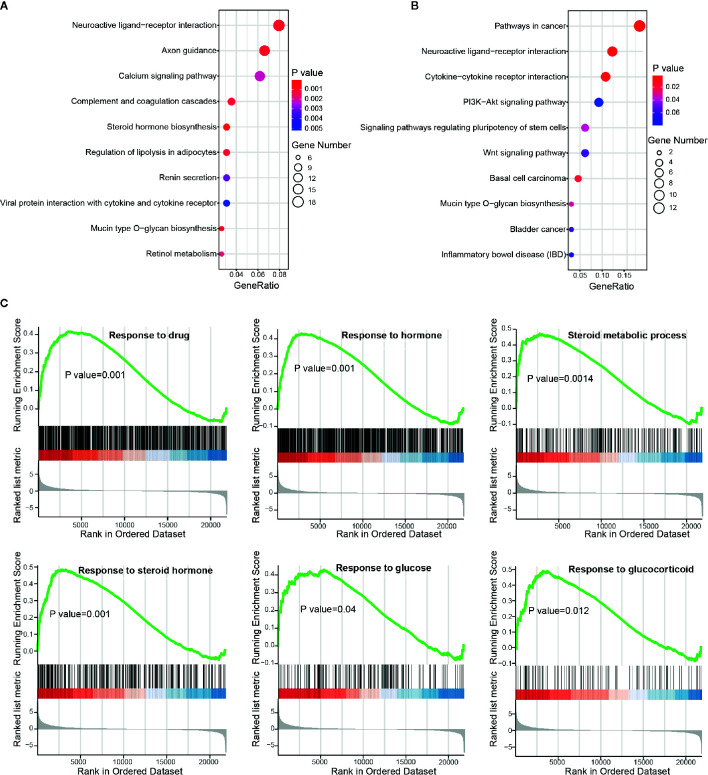
Functional enrichment analysis of differential protein-coding genes. **(A)** Significantly enriched pathways of up-regulated genes. **(B)** Significantly enriched pathways of down-regulated genes. **(C)** Running scores and pre-ranked lists of up-regulated genes in metabolism-related biological processes.

Functional enrichment analysis was next performed to inspect the dysregulated biological processes or pathways that BBD may affect in PDAC (see *Materials and Methods*). DEGs were enriched in cancer-related biological processes, such as “Positive regulation of MAPK cascade”, and cancer-related pathways, such as “Pathways in cancer” and “Wnt signaling pathway” (**Supplementary Figures 5A, B**). To further investigate BBD modulated genes, we performed enrichment analysis separately in up-regulated and down-regulated genes. The up-regulated genes were found to be enriched in metabolism-related pathways, such as “Steroid hormone biosynthesis” and “Regulation of lipolysis in adipocytes” (**Figure 3A**). And down-regulated genes were enriched in cancer-related pathways, such as “Pathways in cancer” and “Cytokine-cytokine receptor interaction” (**Figure 3B**). GSEA analysis revealed that up-regulated genes were significantly enriched in metabolism-related processes, such as “Response to drug”, “Response to hormone”, “Steroid metabolic process”, “Response to steroid hormone”, “Response to glucose”, and “Response to glucocorticoid” (**Figure 3C**), as well as “Steroid hormone biosynthesis” and “C21-steroid hormone metabolic process” (**Supplementary Figure 5C**). And BBD treatment also suppress immune-related processes, such as “Positive regulation of neutrophil migration”, “Regulation of neutrophil migration”, “Regulation of T-helper 17 type immune response”, and “Positive regulation of leukocyte mediated cytotoxicity” (**Supplementary Figure 5D**). Our results suggested that BBD treatment may inhibit tumor growth of PDAC through suppressing cancer pathways and adjusted metabolic processes.

### Tumor-Related Long Non-Coding RNAs Are Modulated by BBD Treatment

**Figure 4:**
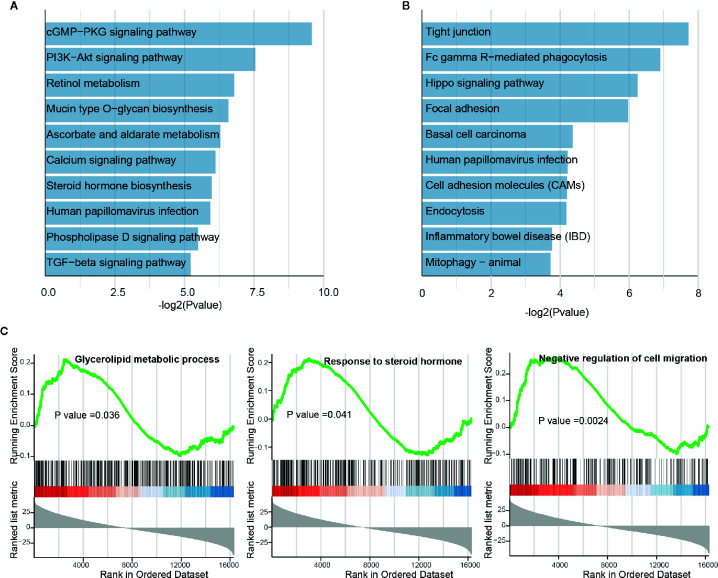
Functional enrichment analysis of differential long non-coding RNAs. **(A)** Significantly enriched pathways of up-regulated lncRNAs. **(B)** Significantly enriched pathways of down-regulated lncRNAs. **(C)** Running scores and pre-ranked lists of up-regulated lncRNA-associated protein-coding genes.

In the differential analysis, lncRNAs were also found to be dysregulated upon BBD treatment. To examine the biological function of BBD affected lncRNAs, we first assigned differential lncRNAs to associated protein-coding genes (see *Materials and Methods*). Enrichment analysis of differential lncRNA-associated protein-coding genes revealed that up-regulated lncRNAs are enriched in metabolism-related pathways or biological processes, such as “Retinol metabolism”, “Mucin type O-glycan biosynthesis” and “Negative regulation of amyloid precursor protein catabolic process” (**Figure 4A** and **Supplementary Figure 6A**). Additionally, down-regulated lncRNAs were found to be enriched in cancer-related pathways and biological processes, such as “Tight junction”, “Hippo signaling pathway” and “Response to cAMP” (**Figure 4B** and **Supplementary Figure 6B**). Up-regulated lncRNAs were revealed to be significantly involved in metabolic processes in the GSEA analysis, such as “Glycerolipid metabolic process” and “Response to steroid hormone” (**Figure 4C**). BBD treatment was also found to elevate lncRNAs associated with process of “Negative regulation of cell migration”. The down-regulated lncRNAs were shown to be involved in metabolic processes of non-coding RNAs, such as “ncRNA metabolic process”, “rRNA metabolic process”, “snRNA metabolic process”, and “tRNA metabolic process” (**Supplementary Figure 6C**). These results suggested that BBD treatment might also modulate metabolism or cell migration processes through targeting lncRNAs in PDAC.

### BBD Regulated Genes Are Clinically Relevant in Pancreatic Cancer

**Figure 5:**
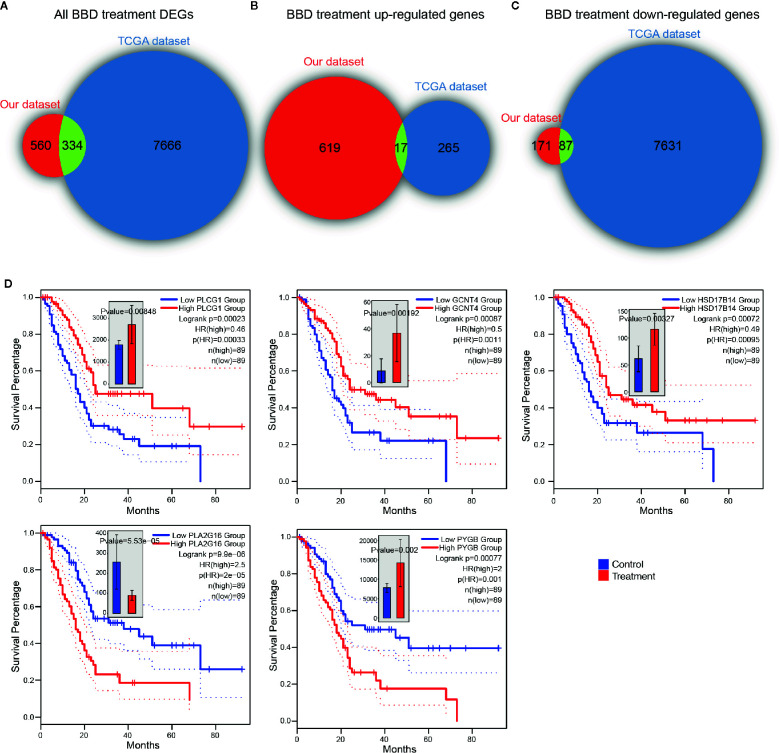
BBD treatment DEGs are clinically relevant in TCGA PAAD cohort. Overlaps of TCGA PAAD differentially expressed genes and all BBD treatment DEGs **(A)**, BBD treatment up-regulated genes **(B)** and BBD treatment down-regulated genes **(C)**. **(D)** Kaplan–Meier survival curves of gene PLCG1, GCNT4, HSD17B14, PLA2G16, and PYGB in TCGA PAAD cohort.

To further inspect the clinical relevance of BBD modulated genes, we investigated their association with patient survival in TCGA PAAD cohort. In TCGA PAAD cohort, 7,718 up-regulated genes and 282 down-regulated genes were found across chromosomes (**Supplementary Figure 7A**). Overall, DEGs in our dataset were significantly overlapped with TCGA differential genes (P value = 2.22e-16, hypergeometric test, **Figure 5A**). Around half of BBD up-regulated genes were down-regulated in the TCGA PAAD cohort (P value = 2.56e-04, hypergeometric test, **Figure 5B**). A substantial percentage of TCGA PAAD down-regulated genes were activated in our BBD treatment samples (P value = 1.70e-05, hypergeometric test, **Figure 5C**). These results suggested that BBD treatment might activate tumor suppressors and inhibit oncogenes in pancreatic cancer, thus suppressing tumor growth. Among all DEGs in BBD treatment samples, 15 DEGs were significantly associated with survival of patients with pancreatic cancer, wherein 13 genes were up-regulated and two genes were down-regulated (**Supplementary Figure 7B**). Among the up-regulation genes, high-expression of metabolic genes PLCG1, GCNT4, and HSD17B14 were associated with longer survival time, while those of PLA2G16 and PYGB were associated with shorter survival time (**Figure 5D**). The low expression of down-regulated gene LY6D was associated with longer survival time, while that of CHRM5 was associated with shorter survival time (**Supplementary Figure 7C**). These findings indicate that BBD treatment may clinically benefit patients with pancreatic cancer through targeting key cancer-related genes.

## Discussion

BBD has been found to be partially effective for patients with pancreatic cancer. However, the underlying molecular mechanism is unclear. Illuminating the possible mechanism will promote the clinical practice of BBD treatment on pancreatic cancer patients. By computational analysis of RNA-seq data of BBD treatment samples, we first presented the gene expression changes after BBD treatment.

Down-regulated genes in the BBD treatment group were found to be enriched in cancer-related pathways, suggesting that BBD may regulate key oncogenes and corresponding pathways to inhibit tumor growth. In addition to activating tumor suppressors, BBD up-regulated genes were enriched in metabolic processes. BBD may adjust cancer induced metabolic dysregulation to prohibit tumor progression. BBD is a fixed powder, containing hundreds of ingredients, which impedes the recognition of effective molecules. Our study paves a way to filter out effective molecules, wherein these identified key genes may be targets.

Besides regulating protein-coding genes, BBD treatment was also found to modulate lncRNAs in PDAC. Our results showed that up-regulated protein-coding genes and lncRNAs were both enriched in metabolism-related pathways. We proposed that BBD treatment regulated metabolic reprogramming through modulating transcription in PDAC.

In the present study, we only analyzed samples from 23.2 g/kg dose BBD treatment group. The group of 23.2 g/kg dose treatment showed the strongest suppression effect on tumor growth of PDAC. Thus, transcriptome in this group was supposed to be the most widely affected by BBD treatment. Our study is the first to present the transcriptional changes of PDAC after BBD treatment. Although we did not perform additional experiments to confirm our findings in larger size of samples, our results provide insights in the underlying molecular mechanisms that BBD suppresses tumor growth of PDAC. Our study will promote the investigation of BBD pharmacological mechanism and propel the optimization of BBD therapeutics for patients with pancreatic cancer.

## Data Availability Statement

The datasets presented in this study can be found in online repositories. The names of the repository/repositories and accession number(s) can be found in the article/**Supplementary Material**.

## Ethics Statement

The animal study was reviewed and approved by Fudan University Shanghai Cancer Center Ethics Committee.

## Author Contributions

LL and LS supervised the study. LS, CP, and XC downloaded the data. LS, XQ, YC, XZ, and ZF performed the computational analysis and visualization. ZF performed the experimental validation. LS, ZF, and LL wrote the manuscript. All authors contributed to the article and approved the submitted version.

## Funding

This study was supported by grants from Natural Science Foundation of Shanghai, China (No.19ZR1411400) and National Natural Science Foundation of China (No. 8187140429).

## Conflict of Interest

The authors declare that the research was conducted in the absence of any commercial or financial relationships that could be construed as a potential conflict of interest.
