# Supplementary material for: BaBao Dan Suppresses Tumor Growth of Pancreatic Cancer Through Modulating Transcriptional Reprogramming of Cancer-Related Genes
Source: Front Oncol. 2020 Nov 19;10:584330. doi: 10.3389/fonc.2020.584330 (PMC7710661; doi:10.3389/fonc.2020.584330)
Supplement: Supplementary file 1 [file DataSheet_1.pdf]

Supplementary Figure S1

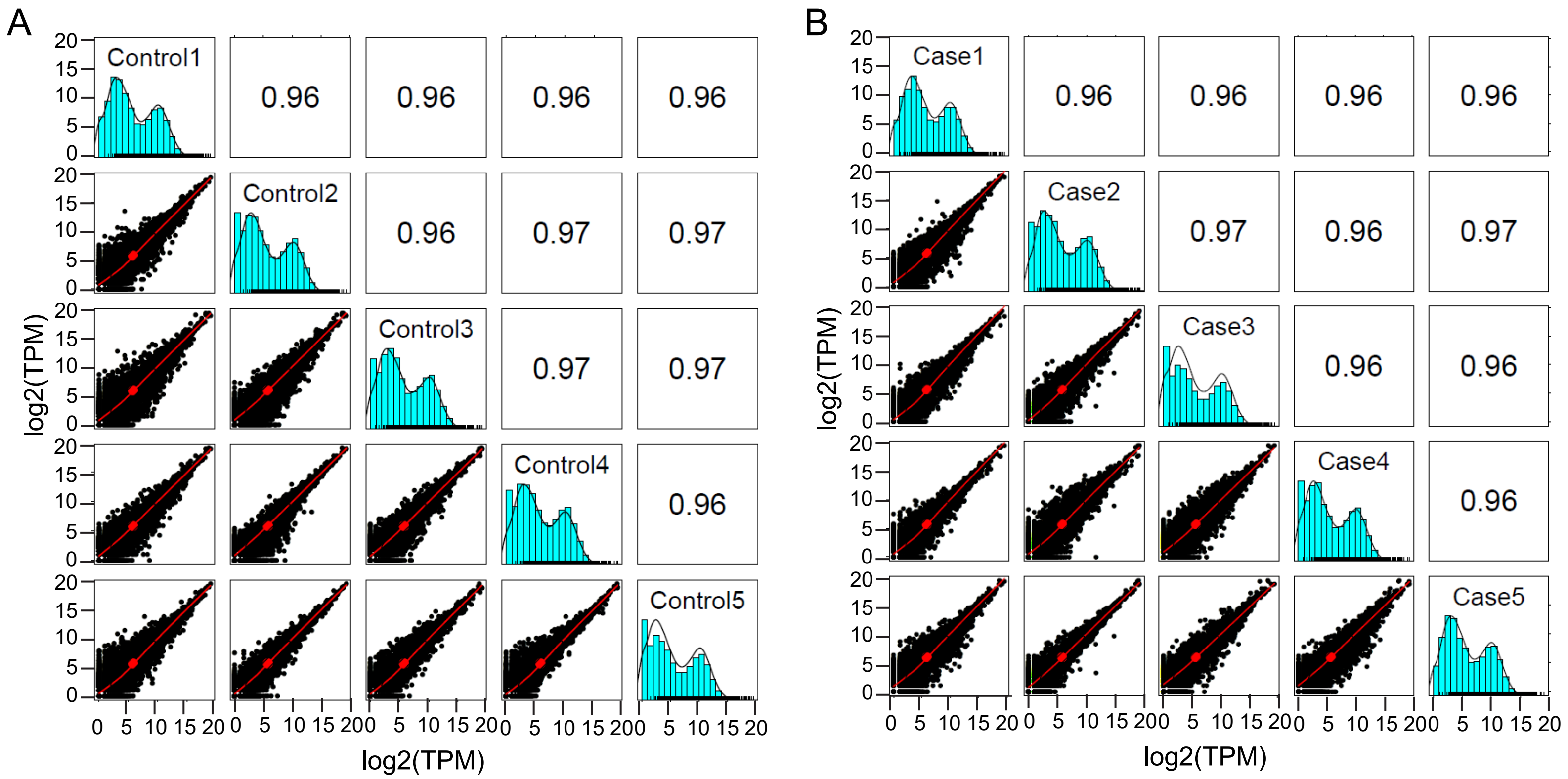

Supplementary Figure S1. Correlations of gene expression between different samples in control **(A)** and 23.2g/kg dose BBD mice groups **(B)**.

Supplementary Figure S2

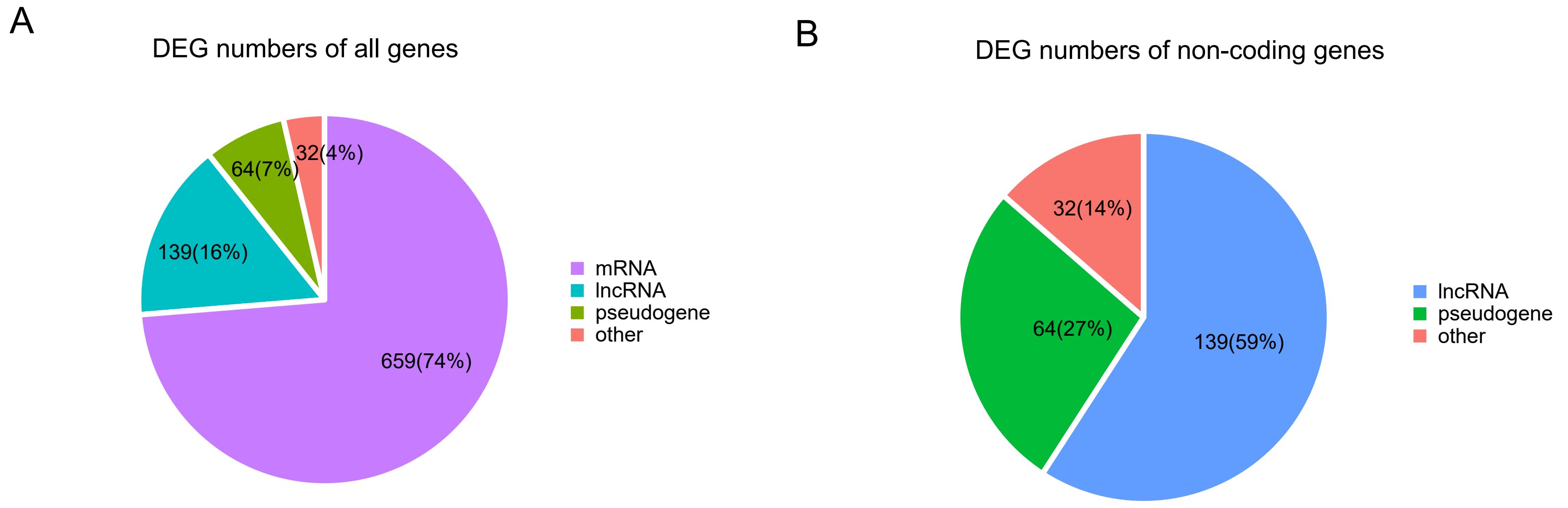

Supplementary Figure S2. Differentially expressed genes in different gene types. (A) DEG numbers and percentages in all gene types. **(B)** DEG numbers and percentages in non-coding gene types.

Supplementary Figure S3

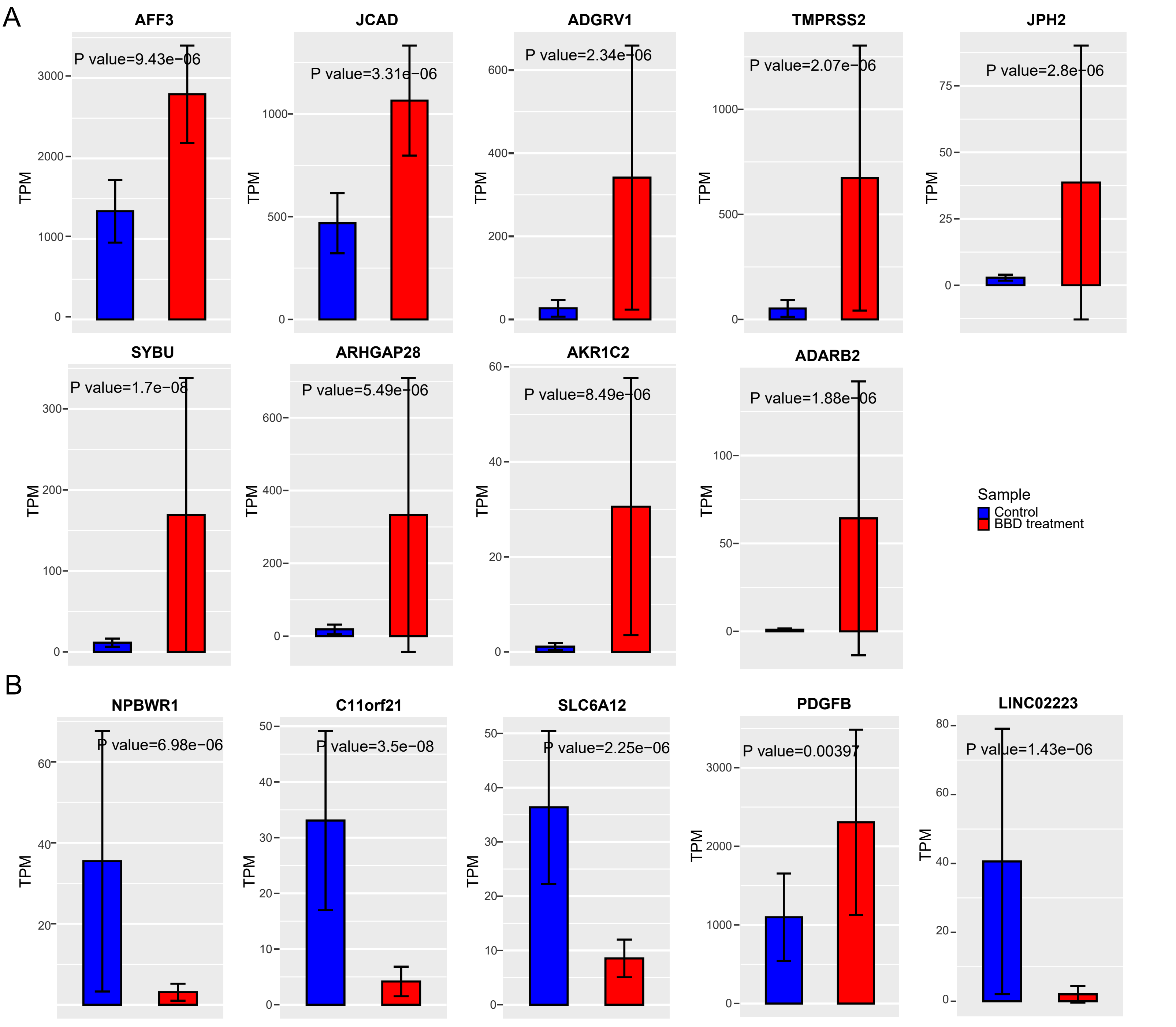

Supplementary Figure S3. Comparisons gene expression of top DEGs between BBD treatment and control groups. **(A)** Comparisons of gene expression of 9 example up-regulated genes between BBD treatment and control groups. **(B)** Comparisons of gene expression of 5 example down-regulated genes between BBD treatment and control groups.

Supplementary Figure S4

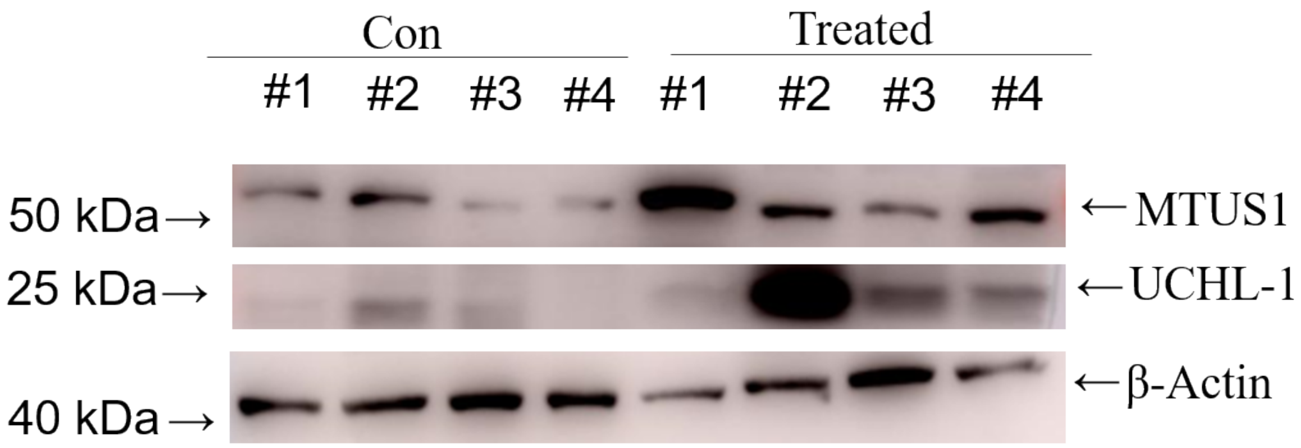

Supplementary Figure 4. Western blotting validation of MTUS1 and UCHL-1 in BBD treatmetn and control samples.

Supplementary Figure S5

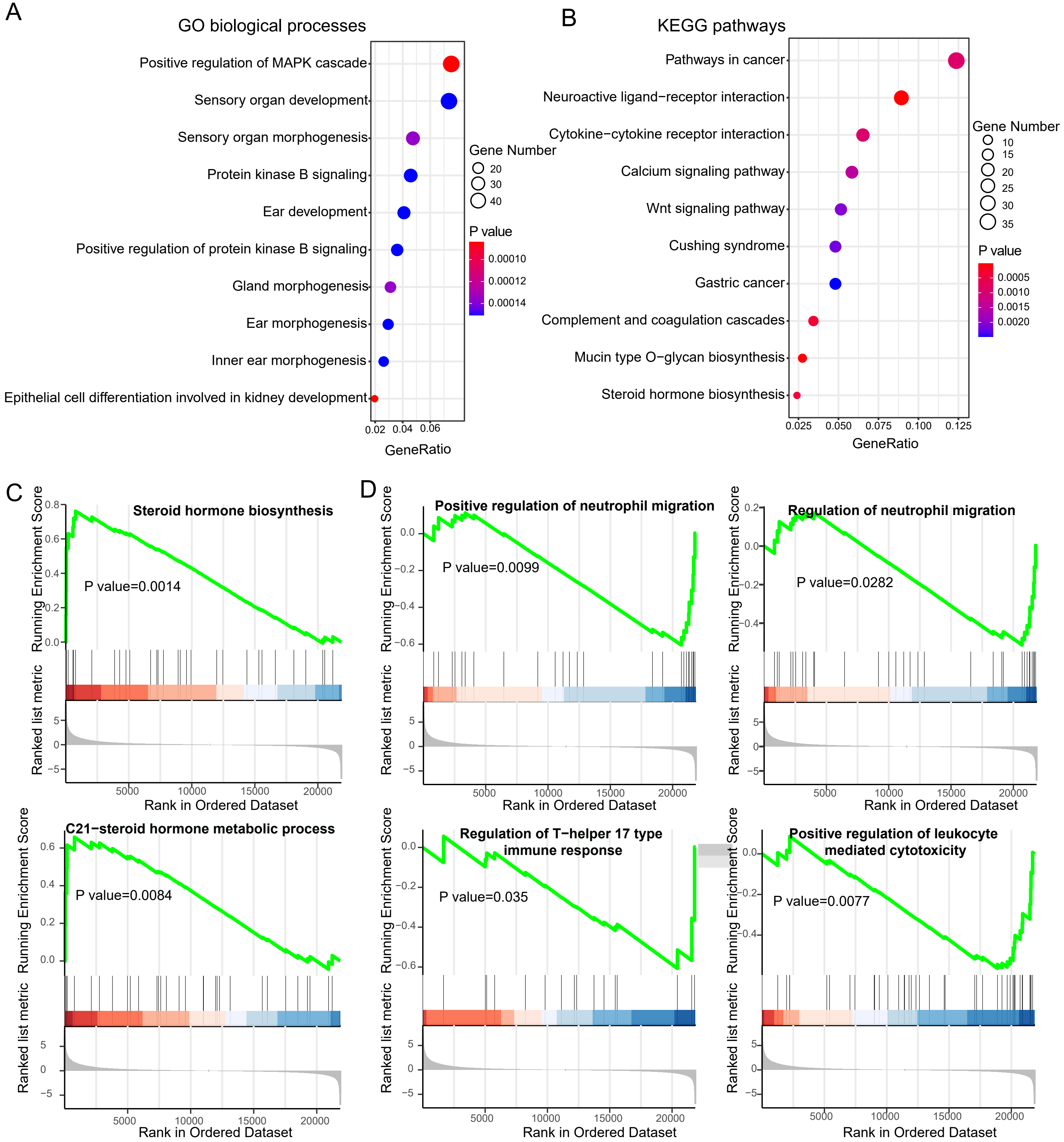

Supplementary Figure S5. (A) Significantly enriched GO biological processes of all differential genes. **(B)** Significantly enriched KEGG pathways of all differential genes. **(C)** Running scores and pre-ranked lists of up-regulated genes in "Steroid hormone biosynthesis" and "C21-steroid hormone metabolic process". **(D)** Running scores and pre-ranked lists of down-regulated genes in immune-related biological processes.

Supplementary Figure S6

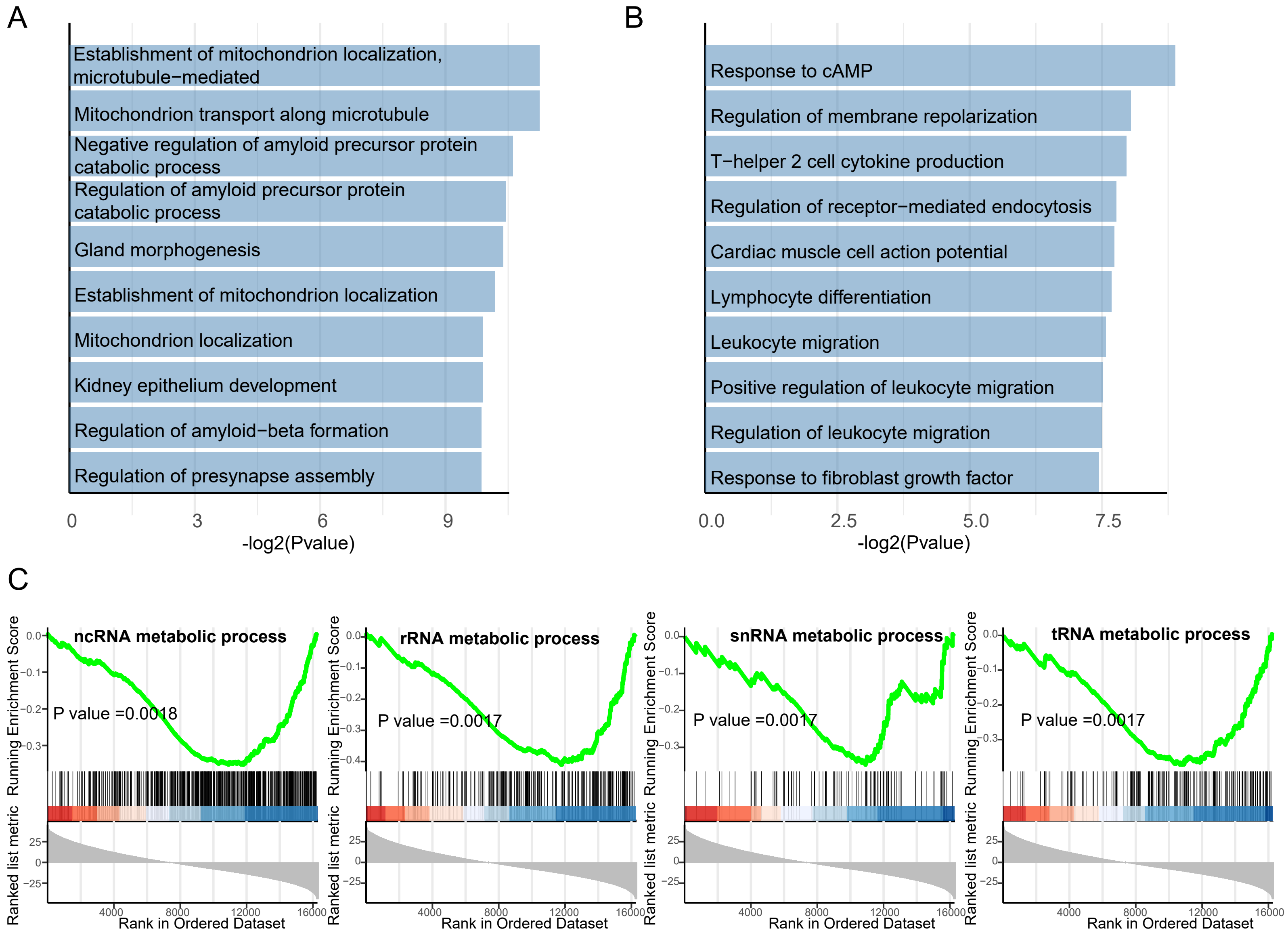

Supplementary Figure S6. Functional enrichment analysis of differential lncRNAs in BBD treatment group. (A) Significantly enriched GO biological processes of up-regulated lncRNAs. **(B)** Significantly enriched GO biological processes of down-regulated lncRNAs. **(C)** Running scores and pre-ranked lists of down-regulated lncRNA-associated protein-coding gnes.

Supplementary Figure S7

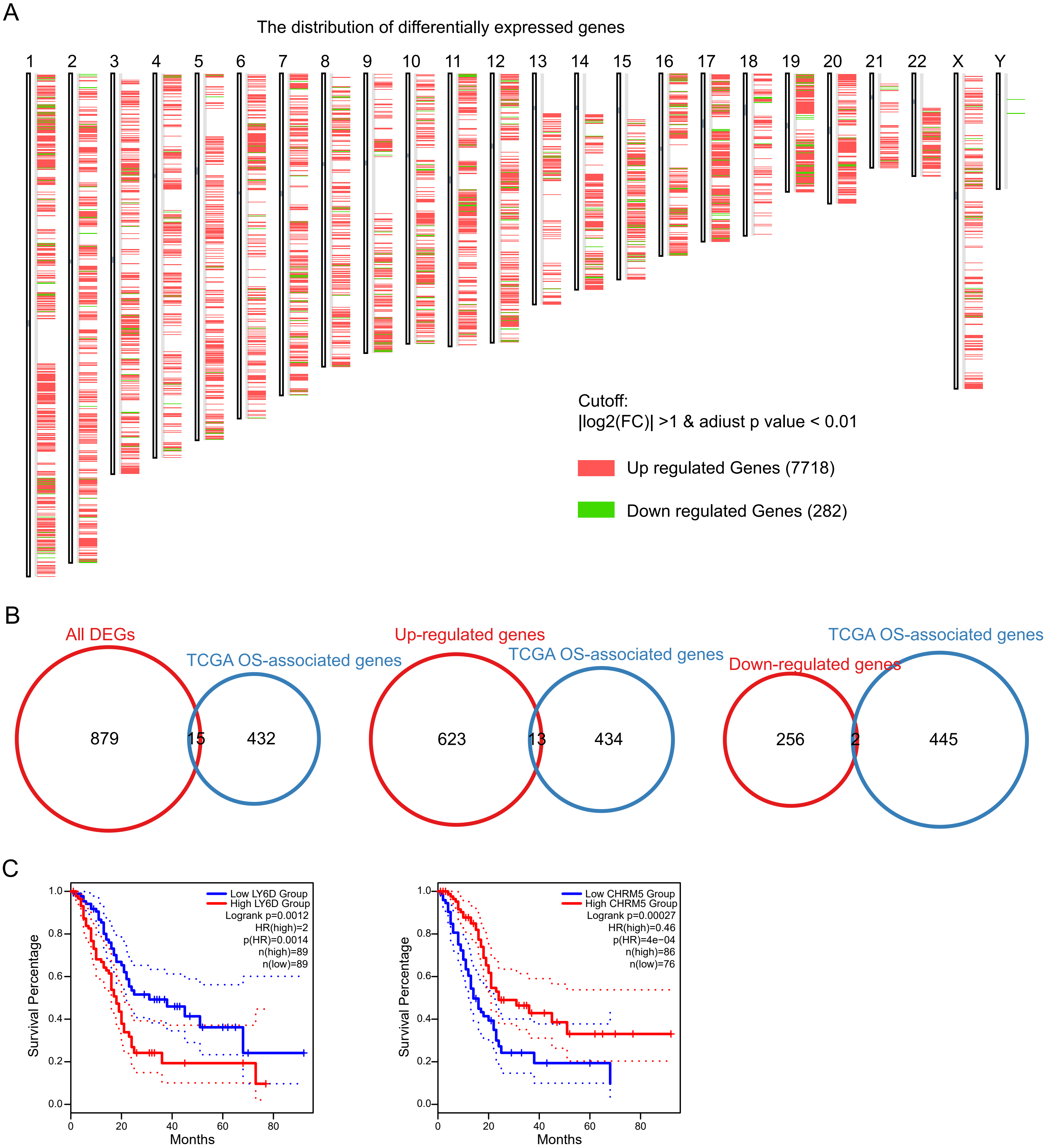

Supplementary Figure S7. BBD treatment DEGs are associated with overall survival of pancreatic cancer patients. (A) The distribution of DEGs in TCGA PAAD cohort acorss chromosomes. **(B)** Overlaps between TCGA PAAD OS-associated genes and all BBD treatment DGEs, BBD treatment up-regulated genes and BBD treatment down-regulated genes, respectively. **(C)** Kaplan Meier curves of LY6D and CHRM5 genes in TCGA PAAD cohort.

Supplementary Figure S8

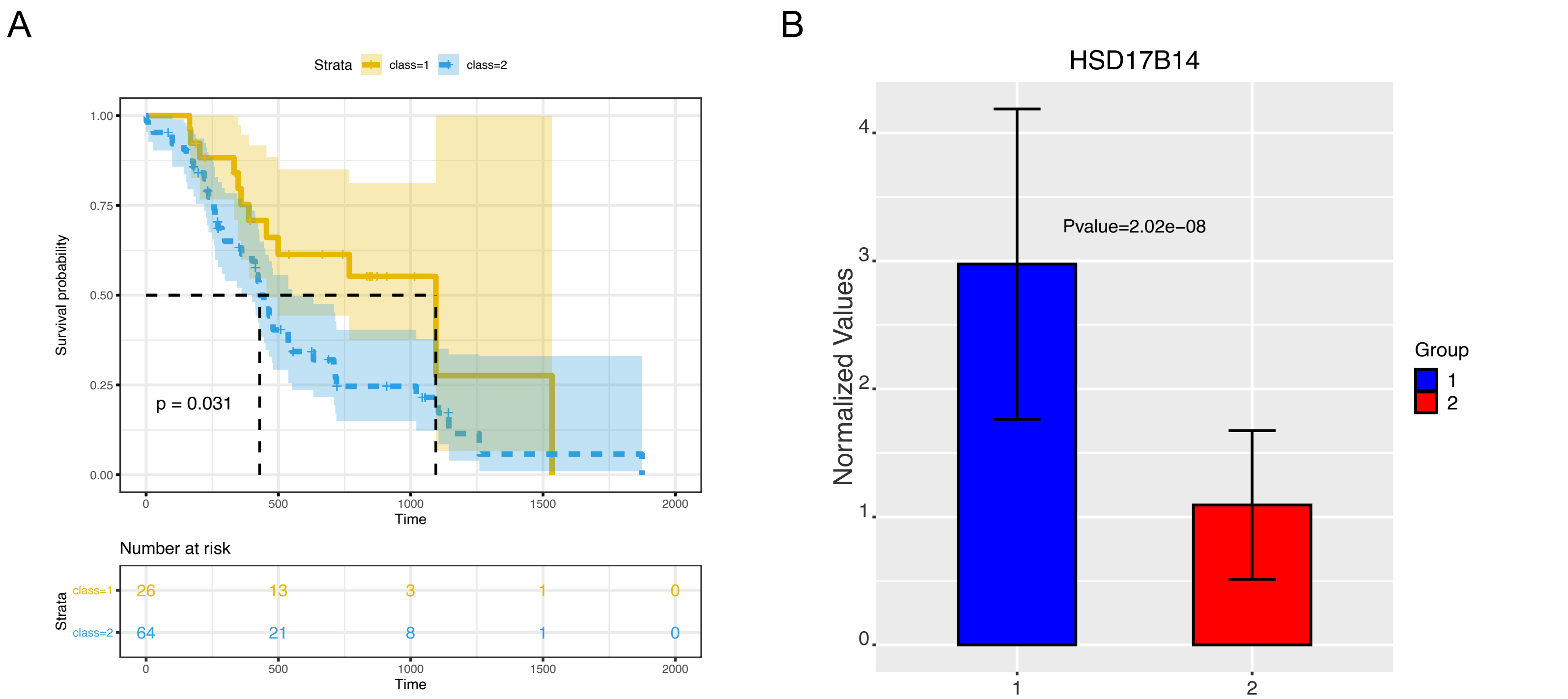

Supplementary Figure S8. The survial curve (A) and expression comparison (B) of HSD17B1 in PACA-AU cohort from ICGC project.
